# A ritual way of ageing well: a qualitative study of a comprehensive, culturally grounded ritual-based health programme for older adults in urban China

**DOI:** 10.3389/fpsyg.2026.1887093

**Published:** 2026-08-19

**Authors:** Xusheng Zhang, Qi Wang

**Affiliations:** 1 Department of Social Work, School of Public Administration, Hangzhou Normal University, Hangzhou, Zhejiang, China; 2 Center on Behavioral Health, Faculty of Social Sciences, The University of Hong Kong, Pokfulam, Hong Kong SAR, China

**Keywords:** Chinese culture, healthy ageing, ritual, urban China, holistic health, qualitative study

## Abstract

**Introduction:**

Rapid population ageing, urban migration, and the weakening of multigenerational households have left many older adults in Chinese cities facing intertwined physical decline, psychological distress, social loss, and a diminished sense of purpose. Conventional responses tend to be specialized and clinical and are often experienced by older Chinese adults as fragmented, unfamiliar, or stigmatizing. Collective ritual, defined as structured group practices using culturally familiar forms, such as greeting, blessing, mutual recognition, coordinated movement, communal singing, embrace, sharing, and role-taking to cultivate connection, emotion regulation, and meaning, is an ancient and culturally embedded practice that has always addressed body, mind, emotion, relationship, and spirit together.

**Methods:**

This study examined how a comprehensive, culturally grounded ritual-based health programme, *“Long and Prosperous Life” (Fushoubainian)*, supports ageing well in urban Beijing. Using a qualitative collective case-study design, we interviewed 21 participants: one programme designer, six frontline social workers, and fourteen middle-aged and older programme participants aged 48–77 years who joined the collective rituals for at least 6 months. Interviews were supplemented by informal follow-up contacts and field engagement after 6 years. We analysed transcripts using reflexive thematic analysis informed by the psychology of ritual and of ageing.

**Results:**

Five interwoven dimensions of benefit were identified: the programme rebuilt belonging and interpersonal trust through a culturally resonant idiom of fictive kinship; it reworked beliefs about ageing, illness, and the self, fostering reappraisal, optimism, and perceived control; it awakened the body, with participants reporting improved mobility, sleep, energy, and health behaviours and better management of chronic disease; it regulated emotion through synchronised movement, communal song, laughter, and embrace; and it restored meaning through contribution, leadership, and, for some, a transcendent reorientation toward gratitude and non-attachment. Crucially, these were not five separate effects but facets of a single ritual practice that integrated physical from mental, individual from collective, and health from culture. Follow-up interview themes were also summarized and reported.

**Discussion:**

We argue that community ritual should be understood not as a standard mental-health intervention but as a comprehensive, low-cost, culturally congruent way of ageing well, and we offer a transferable model for psychologists, gerontologists, and programme designers.

## 1 Introduction

Population ageing is transforming the experience of later life globally, with particularly profound demographic and social shifts observed in China. By the end of 2020, more than 264 million Chinese residents were aged 60 years or older (18.7% of the population), a figure projected to surpass 400 million (roughly one in three citizens) by 2035 (National Bureau of Statistics of China, 2021; Han et al., 2020). This demographic transition has unfolded alongside rapid urbanisation, the internal migration of younger family members, and the gradual loosening of multigenerational living arrangements (Dong et al., 2012). For many older urban residents, the result is a distinctive and many-sided predicament. Physical decline and the onset of chronic disease coincide with the loss of occupational roles, the thinning of kin networks, bereavement, and a culturally salient gap between the filial care older adults expected and the care their busy, geographically dispersed children can provide (Dong et al., 2012; Chen et al., 2022). Physical frailty, loneliness, depressive symptoms, and a diminished sense of purpose tend to arrive together and to amplify one another, and each is a robust predictor of functional decline and mortality in later life (Berkman et al., 2000; Holt-Lunstad et al., 2015).

The challenges of later life are thus comprehensive: they are at once physical, psychological, social, and existential. Yet the dominant responses to them are not. Health and social-care systems characteristically address these strands one at a time and through specialised, often clinical, channels, such as a pharmacological treatment for hypertension, an individual psychotherapy for depression, a meal service or a day centre for isolation. For older Chinese adults in particular, this fragmentation carries a cost. Single-target services can feel disconnected from the way ageing is actually lived; clinical psychological treatment, while effective for late-life depression, reaches only a fraction of those who could benefit, is costly to scale, and is frequently experienced as unfamiliar or stigmatising in a culture where distress is commonly expressed and managed in somatic, relational, and collective rather than clinical terms (Pinquart et al., 2007; Chen et al., 2023; Ho et al., 2014). The World Health Organization’s framing of healthy ageing as the development and maintenance of functional ability and intrinsic capacity across physical and mental domains underscores what is needed: not more single-target services but comprehensive, integrative, and culturally congruent approaches that support the whole person and that work with, rather than against, the social and symbolic worlds older adults already inhabit (World Health Organization, 2015).

Collective ritual is a long-standing human answer to exactly this need for integration. Rituals, defined as patterned, symbolic, and emotionally charged actions performed together, often at fixed times and places are among humanity’s oldest practices for caring for the whole person (Durkheim, 1995; Collins, 2004; Geertz, 2013). Across cultures, ritual has never separated the body from the mind, the individual from the group, or health from meaning: a single ritual practice may move the body, settle the emotions, bind the community, and orient the person toward what matters, all at once. Contemporary psychological research has begun to specify these functions: rituals regulate emotion by imposing structure and a sense of control on uncertainty and loss; they connect people through shared attention and synchronized action; and they help people pursue valued goals and consolidate identity (Hobson et al., 2018; Norton and Gino, 2014). In China specifically, culturally familiar collective body practices, including tai chi, qigong, communal square dancing, group singing, seasonal health customs are accessible, non-stigmatising, embedded in everyday community space, and associated with better physical function, lower depressive symptoms, reduced loneliness, and higher life satisfaction among older adults (Lan et al., 2013; Chen et al., 2022; Lei et al., 2016; Laird et al., 2018). Such practices are not “interventions” delivered to older people; they are ways of being an older person in a shared cultural world.

Despite this promise, three gaps limit current understanding. First, the evidence on ritual-like activities for older adults is overwhelmingly outcome-focused and treats discrete activities in isolation, for example, a study of tai chi here, of group singing there, so that the value of a comprehensive, multi-component ritual practice as an integrated whole is rarely examined (de Diego-Cordero et al., 2022). Second, the experimental psychology of ritual has been built largely from brief, decontextualized laboratory manipulations; how ritual works when it is sustained, collective, and woven into community life over years remains under-studied (Hobson et al., 2018). Third, and most importantly for this paper, when ritual-based programes are evaluated, they are often implicitly assimilated to the template of a standard mental-health or behavior-change intervention, a framing that obscures what may be most valuable about them, including their comprehensiveness, their refusal to compartmentalise health, and their deep cultural embeddedness (Hwang, 2012).

This study addresses these gaps through an in-depth qualitative study of *“Long and Prosperous Life” (福寿百年, Fushoubainian)*, a community-driven, ritual-based health programme for older adults launched in 2015 by a non-governmental organization in Beijing. Rather than asking whether a particular activity “works,” we ask how a comprehensive ritual practice supports ageing well as a whole. Our research questions were: (i) Through what processes do older participants, frontline social workers, and the programme designer understand the programme to affect physical and mental health in later life? (ii) Which ritual components and symbolic practices are experienced as valuable, and why? (iii) How are these processes shaped by, and dependent upon, the Chinese cultural context? In answering them we aim to move beyond a catalogue of beneficial activities toward an account of community ritual as a comprehensive, culturally grounded way of ageing well.

## 2 Conceptual framework: ritual as a comprehensive, culturally grounded practice of ageing well

To interpret participants’ accounts we drew together three literatures: the psychology of ritual, the multidimensional understanding of health in later life, and cultural psychology into a single conceptual framework. We treated this framework as a sensitising lens rather than a fixed coding scheme, remaining open to processes that emerged inductively from the data.

### 2.1 Ritual as an integrative practice, not a single-mechanism technique

A central premise of this study is that ritual should not be reduced to any one of its effects. A growing integrative literature identifies several psychological functions of ritual that ordinarily operate together (Hobson et al., 2018). Rituals regulate emotion: their predictability, repetition, and formal structure impose order on situations of uncertainty, loss, and threat, restoring a sense of control and dampening anxiety (Hobson et al., 2018; Norton and Gino, 2014). Rituals connect people: shared attention, synchronised movement, and joint emotional arousal increase felt similarity, trust, and affiliation, a process classically described by Durkheim as collective effervescence and elaborated by Collins as the generation of “emotional energy” within interaction-ritual chains (Durkheim, 1995; Collins, 2004). Rituals also engage the body, recruiting movement, breath, voice, and touch, and they make meaning, marking transitions and aligning everyday action with cherished significance (Geertz, 2013; Hobson et al., 2018). The defining feature of ritual, and the source of its value for ageing lies in the key point that one practice performs all of these at once. This integrative quality distinguishes naturalistic, multi-component community ritual both from the brief single-function ritual manipulations of experimental psychology and from the single-target logic of clinical intervention.

### 2.2 The many dimensions of ageing well

Ageing well is itself multidimensional. The World Health Organization frames healthy ageing as the maintenance of functional ability across interacting physical and mental domains (World Health Organization, 2015), and psychological science has specified the resources on which it depends. Socioemotional selectivity theory holds that, as the future horizon contracts, older adults prioritize emotionally meaningful goals and relationships, making the affective and relational quality of daily life especially consequential (Carstensen, 2006). Belonging is a fundamental human need (Baumeister and Leary, 1995), and its frustration, such as loneliness, is a potent risk factor for both depression and physical morbidity and mortality in older adults (Holt-Lunstad et al., 2015; Hawkley and Cacioppo, 2010). Agency, including perceived control, self-efficacy, and the satisfaction of the basic needs for autonomy, competence, and relatedness underpins motivation, health behavior, and wellbeing, and is threatened by the role losses of retirement and illness (Ryan and Deci, 2000; Bandura, 1997). Meaning and mattering, referred as a sense of purpose, of being valued and useful, and the capacity to reappraise the self in more expansive terms protect against despair (Ryff, 1989; Steger et al., 2006); gerotranscendence theory proposes that ageing can occasion a shift from a materialistic, self-centred outlook toward greater connectedness and equanimity (Tornstam, 2005), while Erikson framed contribution to others and the achievement of integrity as the developmental tasks of later life (Erikson and Erikson, 1997). Critically, these dimensions, including physical vitality, cognition, emotion, social connection, and meaning are not independent. They co-vary and co-produce one another, which is precisely why a practice that addresses them together may achieve more than the sum of single-target services.

### 2.3 The cultural psychology of ritual and ageing in China

How health and ageing are understood, and how ritual works, are culturally shaped. Chinese cultural traditions are distinctive in their holism. In traditional Chinese medicine and folk health belief, body and emotion are not separate substances but aspects of a single system of qi and circulation, so that “physical” and “mental” health are not naturally compartmentalised (Lan et al., 2013; Laird et al., 2018). In Chinese cultural psychology the self is relationally constituted: identity, emotion, and worth are co-produced within networks of obligation and care, and Confucian ethics frame harmony, filial reciprocity, and lifelong self-cultivation as moral projects (Hwang, 2012; Wei and Li, 2013). Ageing well, in this idiom, is a relational and ethical accomplishment, not merely a biomedical one. Two cultural features are especially relevant here. First, the rapid weakening of co-residence and filial availability has produced not only practical care deficits but a culturally specific wound, which is a felt discrepancy between expected and received intergenerational care, that programes may address by offering surrogate, family-like belonging. Second, China possesses a rich repertoire of secular and seasonal collective practices, from tai chi and square dancing to solar-term health customs, that provide culturally familiar, dignified, non-stigmatising vehicles for ritualised participation. The psychological idioms available to older Chinese adults are syncretic, blending Confucian self-cultivation, Daoist and Buddhist motifs of non-attachment, and globally circulating therapeutic practices; effective programes are likely to be those that adapt and hybridize these resources in culturally resonant ways (Castro et al., 2010).

### 2.4 An integrative framework

Bringing these literatures together, we approached the programme not as a mental-health intervention but as a sustained, collective, culturally grounded ritual practice that could plausibly support ageing well across five interwoven dimensions: social belonging, beliefs about the self and ageing, physical vitality, emotional life, and meaning. The framework’s organizing claim is that the value of community ritual lies in its comprehensiveness and cultural embeddedness: in addressing the whole ageing person, through culturally meaningful forms, within a single practice.

## 3 Materials and methods

### 3.1 Study design

We used a qualitative collective case-study design (Crowe et al., 2011; Yin, 2018) to examine how a comprehensive, ritual-based health programme supports ageing well. A case-study approach was appropriate because the phenomenon of interest of how a multi-component ritual practice is experienced and made sense of by those who design, deliver, and receive it is inseparable from its real-world community and cultural context. Reporting follows the 32-item Consolidated Criteria for Reporting Qualitative Research (COREQ) (Tong et al., 2007).

### 3.2 Setting and the intervention

The study was conducted in urban districts of Beijing, where the non-governmental organization “Organization Y” (anonymised) launched the “*Long and Prosperous Life” (Fushoubainian)* programme in 2015. The programme targets community-dwelling middle and older adults aged 45 and above with the explicit aim of promoting holistic health, including physical, mental, social, and spiritual, as well as ageing well. It was developed by the founder of Organization Y (Mr. Song) on the basis of personal caregiving experience, prior practice with older adults, and existing community services, and it is delivered free of charge in community centers and parks. During the study period it reached approximately 200 older adults at the index site, with affiliated groups in neighboring districts.

The programme is best understood not as a set of separate activities but as a comprehensive ritualized way of life, organized around four core ritual components performed collectively at fixed times and places and led by trained group leaders. The programme consists of 12 health-related education classes and following self or group-based activities on daily basis:

•*Morning exercise*: a sequence of synchronized collective movement explicitly designed, in the programme’s own terms, to “open the meridians and activate qi and blood”: it begins with limb stretching and rhythmic meridian patting, moves through brisk group walking and a finger exercise intended to stimulate the body’s channels, and culminates in a “laughter exercise” and the communal singing of three songs of deliberately ascending emotional intensity, interwoven with spoken self-affirmations of health, youthfulness, and joy.
•*Mindset auto-suggestion*: guided exercises, including a practice the programme calls “intention-setting” (念力设定), in which participants verbalize positive health beliefs and a personal aspiration in order to reshape illness schemas and build expectancy and resilience.
•*Mindfulness-based practice*: sitting meditation, breath awareness, mindful walking, and mindful eating, designed to cultivate present-moment attention and integrated bodily, emotional, and spiritual awareness.
•*The Zero-Limit exercise*: a “clearing” practice adapted from the Hawaiian Ho’oponopono tradition and rendered into Mandarin as the repeated phrases “I am sorry; please forgive me; thank you; I love you,” aimed at releasing negative beliefs and cultivating self-forgiveness and gratitude.

These components were embedded within a structured daily routine of “twelve daily practices” (covering waking, hydration, meals, foot-bathing, and bedtime gratitude), seasonal “solar-term” health activities (such as the communal preparation of health porridges and herbal remedies), symbolic practices including group naming (e.g., “Sunset Angels,” “Vital Elders”), greeting embraces, and WeChat groups, weekly lectures, mutual-aid small groups, and latterly, small groups for the self-management of hypertension and diabetes.

### 3.3 Participants and sampling

We used purposive sampling, supplemented by snowball recruitment, to obtain a maximum-variation sample of stakeholders involved in the programme’s design, delivery, and receipt. The wider “*Long and Prosperous Life” (Fushoubainian)* programme allowed community members to participate flexibly: some attended only selected collective ritual activities or classes, whereas others engaged with all core components. For the present study, we recruited service users who were active participants in the programme, defined as those who had attended the 12 health-education classes and had participated in the subsequent ritual practices for at least 6 months. These practices included both individual self-practices, such as morning self-encouragement, and group-based activities, such as collective gatherings, mutual encouragement, and communication through WeChat groups.

Participants were recruited by distributing invitations during collective ritual activities and health-education classes, followed by snowball referrals from participants and coordinators. Inclusion criteria were: (i) being the programme designer, a social-worker coordinator with at least 6 months of involvement, or a service user aged 45 years or older with at least 6 months of programme participation; and (ii) capacity to provide written informed consent. Exclusion criteria were severe cognitive impairment precluding interview or refusal to be audio recorded.

The final sample comprised 21 participants: one programme designer, six social-worker coordinators, and fourteen older service users (Table 1). Among service users, duration of involvement ranged from 6 months to 9 years. However, because the programme was embedded in everyday community life, the frequency and intensity of engagement varied and were not systematically recorded. For example, some participants practized certain activities individually at home, such as daily self-encouragement, but did not attend every group gathering; others participated intermittently because of health conditions, family responsibilities, including caring for grandchildren, or other personal circumstances. Rather than treating participation as a tightly controlled intervention dose, the study sought to examine how such rituals operated in real-life community settings, where engagement is often fluid and shaped by older adults’ daily lives. Sampling and analysis proceeded iteratively, and recruitment ceased when successive interviews produced no new themes (Guest et al., 2006).

**Table 1:** Participant characteristics (*N* = 21).

Code	Role	Sex	Age (years)	Duration of involvement	Programme components engaged
PD-S	Programme designer	M	62	Since 2015 (founder)	Designed and delivers the 12 health-education classes and the subsequent ritual practices; oversees the seasonal and chronic-disease activities.
SW-P	Social worker/coordinator	F	47	8 years	Coordinates the 12 classes and the morning collective ritual; supports small groups and family engagement.
SW-Q	Social worker/coordinator	F	41	6 years	Coordinates the 12 classes; mutual aid; home visits to housebound elders.
SW-R	Social worker/coordinator	F	39	5 years	Coordinates the 12 classes; co-designs the morning ritual; festivals and seasonal activities.
SW-S	Social worker/coordinator	F	33	3 years	Coordinates the 12 classes; leads the recently formed chronic-disease (hypertension/diabetes) groups.
SW-T	Social worker/coordinator	M	52	7 years	Coordinates the 12 classes; partnerships, festivals, and inter-site outreach.
SW-U	Social worker / coordinator	F	31	2 years	Coordinates the 12 classes; small groups and home visits; supports newer participants.
SU-A	Service user	F	67	5 years	Completed the 12 classes; daily morning ritual; weekly lectures; mutual-aid small group; seasonal porridge.
SU-B	Service user	F	64	4 years	Completed the 12 classes; daily morning ritual; choir; mutual-aid small group; home visits.
SU-C	Service user	F	68	6 years	Completed the 12 classes; daily morning ritual; weekly lectures; mutual-aid small group; values the fixed schedule.
SU-D	Service user	F	72	3 years	Completed the 12 classes; daily morning ritual; Zero-Limit practice; mutual aid and home visits.
SU-E	Service user	F	59	2 years	Completed the 12 classes; most-days morning ritual; selective lectures; childcare commitments.
SU-F	Service user	F	48	1 year	Completed the 12 classes; most-days morning ritual; recent joiner seeking post-illness wellbeing.
SU-G	Service user	F	65	4 years	Completed the 12 classes; daily morning ritual; Zero-Limit practice; choir; mutual aid.
SU-H	Service user	F	70	5 years	Completed the 12 classes; daily morning ritual; the twelve daily self-practices; deep engagement with the spiritual idiom.
SU-I	Service user	F	63	3 years	Completed the 12 classes; daily morning ritual; mutual-aid small group; less involved in lectures and leadership.
SU-J	Service user	F	77	7 years	Completed the 12 classes; daily morning ritual (class monitor); seasonal porridge; monthly charity haircuts and home visits.
SU-K	Service user	M	70	4 years	Completed the 12 classes; daily morning ritual (now co-leads); occasional lectures; mutual aid; first attended in a wheelchair.
SU-M	Service user	F	73	6 months	Completed the 12 classes; most-days morning ritual; trying the twelve daily self-practices; dietary change.
SU-N	Service user	F	71	5 years	Completed the 12 classes; daily morning ritual; small-group leader; peer outreach.
SU-O	Service user	M	55	8 months	Completed the 12 classes; several-days-a-week morning ritual; recent joiner; engagement constrained by work.

### 3.4 Data collection

Data were generated primarily through semi-structured, face-to-face interviews conducted between June and July 2017. These interviews were complemented by follow-up interviews with a subset of participants approximately 6 years later. Thus, “follow-up contacts” refers to repeat interviews with selected participants after the initial data collection, rather than continuous monitoring or systematic recording of attendance, health outcomes, or programme participation across the full six-year period. In total, participants who took part in the follow-up were interviewed twice. The follow-up interviews were used to explore participants’ longer-term reflections on programme involvement, perceived changes over time, continued or discontinued practices, and the ways in which the ritual activities had become integrated into daily life.

Two interview guides were developed: one for the programme designer and social-worker coordinators, and one for service users. Both guides focused on participants’ experiences of the programme components, perceived changes in physical and mental health, the relational life of the group, and the personal and cultural meanings attached to the programme. Sample questions for service users included: “When did you first join this group?” “Which activities impressed you most?” “What benefits, if any, have you experienced?” “Are there any aspects of the programme that you do not like?” “How are these activities related to your daily life?” and “Would you recommend these activities to others, and why?” Questions for the designer and coordinators explored the origins of the programme, its intended aims, the design of ritual components, observations of participant engagement, and perceived challenges in implementation.

Face-to-face interviews lasted approximately 60 min; follow-up interviews were shorter and focused on changes since the first interview. All interviews were conducted in Mandarin Chinese by the first author and three trained social-work research assistants, audio-recorded with consent, and transcribed verbatim. Quotations presented below were translated into English by the first author and backchecked by a second bilingual researcher to preserve psychological and cultural nuance. Field notes documenting non-verbal cues, setting, and reflexive observations were appended to each transcript.

### 3.5 Data analysis

Transcripts were analysed using reflexive thematic analysis (Braun and Clarke, 2006; Braun and Clarke, 2021) in NVivo 15 (Lumivero, Denver, United States), an approach well suited to generating an interpretative, theory-informed account of how a complex practice is experienced. Analysis followed the six recursive phases of reflexive thematic analysis. First, two analysts (XZ and QW) immersed themselves in the corpus through repeated reading of transcripts and field notes. Second, both analysts independently generated initial codes across the full dataset, combining semantic codes close to participants’ language with latent codes capturing underlying processes; coding was approached as an interpretative act rather than a reliability exercise. Third, codes were collated into candidate themes. Fourth, candidate themes were reviewed against coded extracts and the full dataset. Fifth, themes were defined, named, and related to the conceptual framework set out in Section 2. Sixth, the analytic narrative was written, with extracts selected to be vivid and representative. The conceptual framework functioned as a sensitizing resource throughout, and themes were also interrogated for processes that exceeded it. Analytic disagreements were resolved through discussion, and a reflexive audit trail of analytic decisions was maintained.

### 3.6 Trustworthiness and reflexivity

Methodological rigour was pursued through the criteria of credibility, transferability, dependability, and confirmability (Lincoln and Guba, 1985; Shenton, 2004). Credibility was supported by analyst triangulation across professional perspectives, by member reflection (participants reviewed summarised interpretations of their accounts), and by prolonged engagement with the field after 6 years. Transferability was supported by thick description of context, intervention, and participants. Dependability was promoted through a documented audit trail and regular analytic meetings. Confirmability was addressed through reflexive memo-writing: the first author, a sociologist with prior practice experience in Chinese community work, kept a reflexivity journal documenting how personal positionality, including a degree of sympathy for the programme’s ethos shaped engagement with participants and data; the second author, a behavioral-health researcher, acted as a critical interlocutor who interrogated interpretations and guarded against uncritical endorsement of the programme’s own framing.

### 3.7 Ethical considerations

The study received ethical approval from the Research Ethics Committee of Hangzhou Normal University (approval number 15BRK001). Written informed consent was obtained from every participant prior to enrolment. Participants were assured of confidentiality, the voluntary nature of participation, and their right to withdraw at any time without consequence. All identifying information was removed from transcripts and replaced with alphanumeric codes.

## 4 Results

### 4.1 Sample characteristics

The 21 participants comprised one programme designer (male, 62 years; coded PD-S), six social-worker coordinators (five women and one man, aged 31–52 years; coded SW-P to SW-U), and fourteen older service users (twelve women and two men, aged 48–77 years, mean = 65.4 years; coded SU-A to SU-O). Most service users had participated for more than 1 year, and several for 5 years or longer (Table 1).

Reflexive thematic analysis generated five interwoven themes describing how the programme supported ageing well: (1) *a place to belong*—rebuilding trust and relatedness; (2) *reworking beliefs about ageing, illness, and the self*; (3) *awakening the body*—physical vitality and health behavior; (4) *ritual as emotion regulation*; and (5) *mattering, contribution, and the transcendent self*. We present them in sequence for clarity, but emphasize at the outset what the data made unmistakable: participants did not experience these as five separate benefits. They described one practice as a ritualized way of life that touched body, mind, feeling, relationship, and spirit together, and it was this comprehensiveness, realized through culturally familiar forms, that they most valued. The themes and their associated processes are summarized in Table 2.

**Table 2:** The five dimensions of ageing well supported by the ritual-based programme, with associated ritual components and processes.

Theme	Dimension of ageing well	Key ritual components	Processes involved	Illustrative finding
1. A place to belong	Social connection	Greeting embraces; group names; fixed weekly schedule; WeChat groups; mutual-aid small groups	Satisfying the need to belong; repairing interpersonal trust; reducing loneliness through fictive kinship	Strangers in the same apartment block became people who “dissolve each other’s loneliness.”
2. Reworking beliefs about ageing, illness and the self	Cognition and beliefs	Weekly lectures; intention-setting (念力设定); the Zero-Limit “clearing” practice	Cognitive reappraisal; optimism and expectancy; restored perceived control and self-efficacy	Participants moved from “seeing no light at all” to “a little hope.”
3. Awakening the body	Physical vitality	Morning exercise (meridian patting, brisk walking, finger exercise); twelve daily practices; solar-term health activities; chronic-disease groups	Physical activity; improved mobility, sleep and energy; durable health-behavior change; communal chronic-disease management	A wheelchair user came to walk; decades-long insomnia was reportedly resolved.
4. Ritual as emotion regulation	Emotional life	Synchronised morning movement; laughter exercise; communal songs of ascending intensity; embraces; daily structure	Positive-affect induction; behavioral synchrony; structure and predictability as anxiety buffers	“Before, I cried. Now I sing.”
5. Mattering, contribution and the transcendent self	Meaning and spirituality	Group leadership roles; mutual aid and home visits; lectures on love, gratitude and non-attachment	Restored sense of mattering and purpose; the helper-therapy principle; gerotranscendence	“I came to feel that I have my own worth.”

In terms of the weight of each theme, we indicate the extent of its representation across the sample. Theme 1 (belonging) was the most broadly endorsed: all fourteen older participants described loneliness or its repair through family-like warmth and trust, and all six social workers identified the relational work as foundational. Theme 4 (emotion regulation) was endorsed almost as widely: thirteen of fourteen older participants described affective shifts produced by the morning exercise, communal songs, or the greeting embraces, and all social workers commented explicitly on the design’s affective architecture. Theme 3 (awakening the body) was endorsed by eleven of fourteen older participants, those who reported physical change typically also reported behavior change and by all social workers, including those latterly leading the chronic-disease groups. Theme 2 (reworking beliefs) was endorsed by ten of fourteen older participants and explicitly framed by the programme designer; for two of those participants, who had entered the programme in psychological crisis, this work appeared to be the central source of change. Theme 5 (mattering and contribution) was endorsed by twelve of fourteen older participants; the more transcendent reorientation it can occasion was specifically articulated by a smaller subset of five long-term participants and by the designer. We use these counts not to claim quantitative representativeness, which qualitative work cannot deliver, but to make explicit which themes describe widely shared experience and which describe a smaller subset.

### 4.2 Theme 1: A place to belong—rebuilding trust and relatedness

The starting point for most older participants was relational impoverishment. Several had moved to Beijing late in life to help raise grandchildren, leaving behind the dense networks of a lifetime; others had been widowed or lived alone in apartment blocks where neighbors were strangers. One woman who had relocated from north-east China described the affective texture of this loss plainly:

*“When I first came to Beijing, the strongest feeling was loneliness: all my relatives and friends were back home, and here it was just the two of us (self and husband).” (SU-I, female, 63)*

This relational deficit was culturally specific. Participants did not simply miss company; they registered a gap between the close, family-like sociality they had expected in later life and the thin, transactional contact actually available to them in the city. The programme’s first achievement, in participants’ accounts, was to fill this gap with a form of fictive kinship. Older adults repeatedly described the group not as a class or a club but as a family:

*“When we are all together it is just like being a family. I had been ill—I am a cancer patient—and here, with everyone, I am not alone.” (SU-F, female, 48)*

Belonging of this depth had to be built, because it began in suspicion. Many older adults had been targeted by health-product scams and approached a free programme with wariness. Trust developed incrementally, as participants tested the organization’s motives against its conduct over months:

*“We were suspicious at first—free? Why? But we saw that they did not lie to us, they did not sell us anything. So we began to trust them.” (SU-K, male, 70)*

Three features of the programme converted suspicion into belonging. The first was the reliability of embodied co-presence: a fixed time and place, week after week, gave the relationship a predictability experienced as both professionalism and care that “*we know that on Tuesday morning we will see one another*” (SU-C, female, 68). The second was the symbolic and bodily marking of the bond: greeting embraces, group names such as “Sunset Angels,” and continuous WeChat contact made the relationship visible, nameable, and portable into everyday life; the embrace in particular was experienced as a direct transmission of care, with participants speaking of feeling “the love flowing through the body” (SU-K). The third was the dissolving of mistrust between participants themselves, as strangers in the same apartment block became people who greeted, telephoned, and watched (take care of) over one another:

*“We dissolve each other’s loneliness. Everyone cares about each other—at holidays, at birthdays, and when someone is ill.” (SU-B, female, 64)*

This relational repair was the foundation on which every other benefit rested. It satisfied the fundamental need to belong (Baumeister and Leary, 1995) and attenuated the loneliness that is so corrosive to both mental and physical health in later life (Holt-Lunstad et al., 2015; Hawkley and Cacioppo, 2010)—and it did so through a culturally resonant idiom, the family, that gave the new bonds immediate emotional legitimacy.

### 4.3 Theme 2: Reworking beliefs about ageing, illness, and the self

A distinctive feature of the programme was its deliberate work on participants’ beliefs. The designer articulated an explicit model of change:

*“Beliefs shape thoughts, thoughts guide action, and action produces the outcome. So we begin by changing beliefs.” (PD-S)*

This model was operationalized through ritualized practice. Weekly classes challenged the assumption that ageing is necessarily a slide into illness, dependency, and worthlessness. The “intention-setting” practice asked each participant to articulate a positive health aspiration, functioning as a structured exercise in optimism and expectancy. The Zero-Limit “clearing” practice gave participants a portable cognitive tool for interrupting rumination and self-blame, which they described in strikingly practical terms:

*“When you are about to lose your temper, you think of those words—I am sorry, please forgive me, thank you, I love you—and your heart settles.” (SU-G, female, 65)*

*“We say it three times every morning … you speak out all your troubles, and your heart eases at once.” (SU-D, female, 72)*

A recurrent organising belief was the principle the programme summarised as “*I am the source of everything*”—an injunction to locate the origins of one’s distress, and the leverage for change, within oneself. While such a radical internal attribution carries risks, participants generally experienced it as empowering: it reframed passive suffering as something they could act upon and relocated control inside the self at a stage of life when control is widely felt to be slipping away (Ryan and Deci, 2000). The reported consequences were consistent: a shift from catastrophic to benign interpretations of one’s own ageing and illness, and a reduction in habitual anger and rumination that “*my biggest change is that my heart has opened up; I no longer get angry easily*” (SU-G). The most striking accounts came from participants who had entered in crisis. One woman, widowed and living with cancer, had lost all hope before joining:

*“I had stopped seeing any light at all … Through these people I came to see that there are still loving people in the world, and I began to see a little hope.” (SU-H, female, 70)*

For her, the reappraisal of the social world and the reappraisal of the self were inseparable. The programme functioned as a structured, collective platform for cognitive reappraisal, supplying culturally sanctioned schemas for ageing and illness, portable techniques for interrupting maladaptive cognition, and an attributional frame that restored perceived control. The authority of the charismatic “teacher” and the repeated, ritual form in which these messages were delivered were themselves part of the mechanism, lending the new beliefs a social weight that no leaflet could.

### 4.4 Theme 3: Awakening the body: physical vitality and health behavior

What older adults described as the programme’s distinctive value was not work on the mind alone but a refusal to separate mental change from physical change. The shifts in belief and mood that organized Themes 2 and 4 were, in participants’ own accounts, inseparable from concrete, embodied transformations, such as steadier balance, deeper sleep, lower medication use, recovered mobility that generated by the same daily ritual rather than added to it. Treating the programme as a “psychological intervention” (an activity aimed primarily at thoughts and feelings) would therefore misread what older adults said held them: a single, recurring practice that in the space of an hour moved the body, lifted the mood, and rehearsed new ways of understanding the self. Participants spoke first and often of physical change. The morning exercise was designed, in the words of a social worker, around a holistic and culturally grounded logic of the body:

*“The morning exercise opens the meridians and activates qi and blood. First you stretch, then through stamping and patting you rouse the blood, then you raise the body’s energy—'I am healthy, I am young’.” (SW-R, female, 39)*

The reported physical effects were tangible and were what first held many participants. A man who had had a stroke and first attended in a wheelchair traced a bodily trajectory:

*“I came pushed in a wheelchair, and now I can walk. The exercise is mainly good for the bones … and my mood has brightened too.” (SU-K, male, 70)*

Others described improvements in mobility, joint comfort, and everyday stamina, and importantly that linked them to durable changes in health behavior:

*“My joints feel lighter; it is better for my bones. And I have changed my diet—lighter food, less salt.” (SU-M, female, 73)*

*“In a whole year I did not catch a cold once, and I did not take a single pill … I used to drink beer; after the lectures I stopped.” (SU-J, female, 77)*

Sleep, a pervasive concern in later life, recurred as a domain of marked change. The structured “twelve daily practices,” which is a ritualized ordering of waking, hydration, meals, foot-bathing, and bedtime were credited with restoring rest that had been disturbed for decades:

*“I had been an insomniac for decades. After a few months of following these practices, my long sleeplessness was healed.” (SU-H, female, 70)*

These physical benefits were not incidental by-products of a “mental” programme; they were produced by the same ritual architecture and were experienced as one with the emotional and relational changes. The programme’s cultural framing was central here. Its account of the body in terms of meridians, qi, and seasonal balance, its solar-term health customs, and its structured daily regimen drew on a Chinese holistic tradition in which physical and mental health are not separate ledgers but aspects of a single, cultivable vitality (Lan et al., 2013; Laird et al., 2018). Latterly, the programme also formed small groups for the collective self-management of hypertension and diabetes, in which participants reported reducing medication under medical guidance as an extension of the same logic from general vitality toward the ritualised, communal management of chronic disease. That older adults could speak in one breath of lighter joints, deeper sleep, a steadier temper, and a brighter mood is itself the central finding of this theme: the ritual worked on the whole person at once.

### 4.5 Theme 4: Ritual as emotion regulation

Alongside the awakening of the body ran an equally concrete regulation of emotion. The programme’s embodied rituals were experienced as immediately effective, accessible tools for shifting affect, and tools that could be used daily, required no clinical contact, and worked even on mornings that began badly. The morning exercise layered several emotion-regulatory ingredients: synchronised collective movement, rhythmic patting, a “laughter exercise,” and three songs of ascending emotional intensity. A social worker described the deliberate architecture of arousal:

*“It is not just moving the arms and legs. There is the patting, then the inputs—'I am healthy, I am young’—then the big laugh, and then the songs, whose energy keeps rising.” (SW-R, female, 39)*

Participants experienced this sequence as a reliable transformation of feeling. The contrast they drew was between two emotional states of the self:

*“I wake up feeling happy every morning. Before, I cried. Now I sing.” (SU-C, female, 68)*

The movement from crying to singing recurred across interviews and captures the theme precisely: the ritual did not argue participants out of dysphoria; it moved them out of it, bodily and collectively. Synchronized movement and joint singing generated shared positive arousal, the greeting embrace added affiliative touch, and the laughter exercise manufactured positive affect directly. A social worker recounted how the songs reached even acute grief:

*“One woman had lost her husband and could not come out of her grief. When she heard our morning song, she felt it sang straight into her heart—and that is when she joined us.” (SW-S, female, 33)*

Two features amplified the effect. The first was structure: the fixed daily routine and the unvarying sequence of the morning ritual gave shape and predictability to time that, for retired and bereaved participants, could otherwise feel formless such that an imposed order that, as ritual theory predicts, was itself calming (Hobson et al., 2018; Norton and Gino, 2014). The second was cultural familiarity: the programme’s embodied practices resembled the collective morning exercise, tai chi, and group singing already woven into Chinese public life (Chen et al., 2022; Lei et al., 2016), so that participants encountered them not as an alien therapy but as a recognizable, dignified way of being an older person in shared space. Even the imported Zero-Limit practice was naturalized, its Hawaiian origin dissolved into a Mandarin idiom of gratitude and apology consonant with Confucian and Buddhist sensibilities. This cultural congruence allowed the rituals to function as everyday, non-stigmatizing emotion regulation rather than as treatment.

### 4.6 Theme 5: Mattering, contribution, and the transcendent self

The final dimension concerned meaning. Many participants had entered later life with what we came to characterize as a corroded sense of purpose: a felt diminution of being needed, useful, or directed by anything in particular. This was not an interpretive imposition. We coded as evidence of this state participants’ own descriptions of their pre-programme life as “killing time” or “just passing time” (e.g., SU-D’s recollection that she “*had come here to pass the time*”); accounts of waking with no reason to get up; statements that they had stopped expecting anything from the next stage of life; and most starkly, in SU-H’s case that the expectation of dying without anything meaningful left to do. Three biographical patterns clustered with this state: recent retirement that had stripped occupational identity; the maturation of grandchildren that had ended the role of primary carer; and the loss of a spouse. Against this backdrop the programme’s countermove was striking: it offered not entertainment but graduated responsibilities: class duty, group leadership, mutual-aid visits, peer mentorship in the chronic-disease groups that participants experienced as a renewed claim on their time and attention. Theme 5 thus emerged from the contrast between two states they themselves described: the prior absence of being needed and the new experience of mattering, embedded in roles concrete enough to organize the week. These opportunities were graduated and genuine. Engaged participants became group leaders who had “*to take responsibility for everyone: to remember birthdays, to call when someone is sick*” (SU-N, female, 71); others cooked for housebound peers, joined mutual-aid groups, or visited isolated older neighbors. The psychological yield of contribution was named directly:

*“I came to feel that I have my own worth. Before, I just thought I had come here to pass the time.” (SU-D, female, 72)*

The same dynamic operated for the most depleted participants: the stroke survivor who arrived in a wheelchair described moving from receiving help to giving it that “*I have become willing to help others*” (SU-K). This is the helper-therapy principle in a culturally Chinese key: contribution was experienced not as a burden discharged but as a source of one’s own renewal. The designer made the logic explicit that “*we do not do this because others need help; it is our own inner growth that needs it*” (PD-S). For some participants, contribution opened onto something more expansive: a reorientation of the self-articulated in spiritual and philosophical terms. The programme’s vocabulary of love, gratitude, and “non-attachment” gave older adults a culturally available language for late-life development:

*“We sing every morning to value and attain the original state of non-attachment.” (SU-B, female, 64)*

The designer described the intended arc of this development as a movement “*from the small self to the larger self, to the self that no longer grasps*” (PD-S). This progression from a self-defined by grievance and fear toward one defined by connectedness, acceptance, and care for others corresponds closely to gerotranscendence (Tornstam, 2005) and to Erikson’s account of integrity and generativity as the achievements of later life (Erikson and Erikson, 1997). Notably, the social workers described undergoing a parallel transformation, several speaking of the work as a process of “*clearing, purifying, and elevating*” their own outlook (SW-S), which underscored that the programme’s processes circulated through the whole ritual community rather than being confined to its older clients.

### 4.7 Continuities and shifts over time

Because participants’ involvement spanned from 6 months to 9 years, and because we maintained contact with a subset of informants and the field after 6 years, the analysis attended explicitly to whether and how the meaning of participation evolved. Three patterns were evident. First, longer-term participants described a common entry trajectory: an initial period (typically the first three to 6 months) dominated by suspicion and physical curiosity, in which the morning exercise was the primary point of contact and trust remained provisional; a middle period in which embodied benefit and the warmth of group life consolidated commitment; and a later period, reached by those involved for 2 years or more, in which a vocabulary of meaning and contribution moved to the center and participants began to take leadership roles. Second, the meaning of the same ritual deepened with repetition. The greeting embrace, initially registered as friendly, was later described as a “transmission of love”; the morning songs, initially heard as pleasant, became, in SU-H’s words, what “*sang straight into the heart*” in the wake of bereavement. This deepening is consistent with the layered, accrual-by-repetition character of ritual itself, in which felt significance is built through participation rather than delivered at first encounter (Hobson et al., 2018). Third, follow-up contacts revealed that the wider organization contracted between 2018 and 2023, with staff departures and reduced reach in some districts. Participants’ responses to this contraction differed instructively. Those who had moved into the later, contribution-centered phase typically continued the morning ritual and small-group activities on their own initiative: *“they want to withdraw but we cannot; we still insist on doing it*” (SU-N), while more recent participants reported reduced attendance once staff presence weakened. The temporal dynamics thus matter both for understanding how individual change unfolds and for designing programes that can survive the inevitable thinning of charismatic leadership.

## 5 Discussion

### 5.1 Principal findings

This study set out to understand how a comprehensive, culturally grounded ritual practice supports ageing well. Drawing on the accounts of older participants, frontline social workers, and the programme designer, we identified five interwoven dimensions of benefit. The programme rebuilt belonging and interpersonal trust, converting suspicion into a culturally resonant fictive kinship that buffered loneliness; it reworked beliefs about ageing, illness, and the self, supplying schemas and portable techniques for cognitive reappraisal and restoring perceived control; it awakened the body, with participants reporting improved mobility, sleep, energy, health behaviors, and chronic-disease management; it regulated emotion through synchronized movement, communal song, laughter, and embrace; and it restored meaning through contribution, leadership, and, for some, a transcendent reorientation of the self. The decisive finding is not any one of these dimensions but their inseparability. Participants did not report five effects; they reported one ritualized way of life that worked on body, mind, feeling, relationship, and spirit at once.

### 5.2 The value of comprehensiveness: ritual as a way of ageing well, not an intervention

We therefore argue that community ritual of this kind should not be understood as, or assimilated to, a standard mental-health or behavior-change intervention. Conventional interventions are defined by a single target and a single mechanism, such as a therapy for depression, an exercise prescription for frailty, a befriending scheme for loneliness. The programme studied here is defined by the opposite property: a single practice that addresses many targets simultaneously and treats them as one. Its value lies precisely in this comprehensiveness. Three implications follow. First, comprehensiveness is psychologically efficient: because the dimensions of ageing well co-produce one another: belonging makes belief change credible, regulated emotion makes contribution possible, a revitalized body lifts mood, a practice that engages them together can yield broad change that siloed services cannot. The morning exercise alone is at once a belonging ritual, an emotion-regulation device, a programme of physical activity, a belief-rehearsal vehicle, and an arena for contribution. Second, comprehensiveness is what makes the practice a way of life rather than an appointment: it is enacted daily, in community space, by older adults themselves, and so escapes the reach, cost, and stigma limits of clinical provision. Third, to evaluate such a programme with the instruments of a single-target trial would be to measure precisely what is least distinctive about it and to miss what older adults said they valued most.

These findings sit at the intersection of three established literatures and extend each. First, they corroborate experimental, ethnographic, and cross-cultural work showing that collective ritual is a potent vehicle for social bonding, identity formation, and emotion regulation (Durkheim, 1995; Collins, 2004; Hobson et al., 2018; Norton and Gino, 2014; Whitehouse and Lanman, 2014). Whitehouse and colleagues’ synthesis of work on synchronous and emotionally arousing collective ritual identifies “identity fusion,” which is a deep felt union of personal and group selves achieved through shared embodied experience, as a key mechanism through which ritual binds participants to one another and to a common project (Whitehouse and Lanman, 2014). Our data show how this mechanism operates not in a discrete ceremonial event but across years and in ordinary urban community space, and how it is the felt basis of the family-like belonging older adults repeatedly named. Second, they extend the literature on community-based participatory wellbeing programes for older adults (Hikichi et al., 2015; Wallerstein and Duran, 2010). The JAGES Taketoyo study demonstrated that a community programme designed to increase social interaction reduced functional disability and slowed health decline among Japanese older adults (Hikichi et al., 2015); community-based participatory frameworks more broadly emphasize that wellbeing is produced through the partnership of community members and external supporters rather than delivered to them (Wallerstein and Duran, 2010). The present case suggests that the active ingredient in such programes may be precisely the integrative density of ritual that several psychological and social mechanisms acting through one recurring practice, rather than the additive sum of separately delivered components, and helps explain why community-based programes that engage older adults as co-producers tend to outperform service-style provision in retention and reach. Third, the findings speak to the substantial body of work on religious and quasi-religious involvement and later-life wellbeing, which has consistently linked attendance, embodied collective practice, and shared meaning to lower loneliness, better cognitive functioning, and reduced mortality in older adults (Koenig, 2012; Hill et al., 2006). We extend this work by specifying, in a secular Chinese ritual context, the lived processes of belonging built through patient trust-testing, meaning rebuilt through graduated contribution, the body re-engaged through one shared morning practice through which structured collective practice may produce analogous effects without invoking explicitly religious commitment.

### 5.3 The cultural grounding of ritual’s effectiveness

Equally central is the finding that the programme’s effectiveness was inseparable from its cultural form. The family metaphor that legitimized new bonds, the construal of the body in terms of meridians, qi, and seasonal balance, the secular collective body practices that made participation dignified rather than stigmatizing, the authority of the “teacher,” and the syncretic spiritual idiom that hybridized Confucian self-cultivation, Daoist and Buddhist non-attachment, and an imported Hawaiian practice, each of these was not decorative packaging but part of the working mechanism. The programme’s holism is itself culturally specific: it could refuse to separate physical from mental health because the Chinese folk and medical traditions it drew on never separated them in the first place.

It is therefore worth specifying why culturally embedded practices influence engagement, meaning making, and wellbeing, which is a question that our data converge with three distinct bodies of literature. At the level of engagement, cultural familiarity lowered the threshold for participation: square-dancing-like morning movement, communal porridge at solar terms, and the form of the greeting embrace were already legible to older Chinese adults, who could enter them without the identity-cost that signing up for a “therapy” would have carried. This is consistent with sustained findings that culturally adapted health and wellbeing interventions achieve higher uptake, retention, and adherence than culturally generic ones precisely because they are encountered as continuous with everyday life rather than as alien expertise (Castro et al., 2010; Bernal et al., 2009). At the level of meaning-making, the programme’s idioms, such as family, harmony, self-cultivation, non-attachment were neither invented nor imported wholesale; they drew on Confucian, Daoist, and Buddhist scripts that already organise what it means to age well in Chinese cultural psychology (Hwang, 2012; Wei and Li, 2013). Cultural-psychological work has long shown that interpretive frameworks for selfhood, suffering, and ageing differ systematically across cultures and that wellbeing depends in part on the availability of culturally legitimate templates for late-life identity (Hwang, 2012); participants in the present study placed their experiences within available collective stories, such as being a “sister,” being on the path of “clearing,” returning to the “original state of non-attachment,” and thereby made them coherent. At the level of wellbeing itself, the programme’s refusal to compartmentalize body, mind, and relationship was made possible by Chinese medical and ethical traditions in which qi, emotion, and conduct are conceptually continuous (Lan et al., 2013; Laird et al., 2018).

Western psychological theories built on assumptions of categorial separation between cognition, affect, and the body can struggle to capture the integrated kind of change the programme produced; the Chinese holistic frame did not need to assemble these elements because it had never analytically split them. Taken together, these three levels imply that cultural grounding is not a final localizing adjustment to a universal intervention template, but constitutive of how community ritual produces engagement, meaning, and wellbeing. programes elsewhere should be designed to mobilize, not merely to translate into the host culture’s own resources for ageing well, and psychology and gerontology stand to learn from cultural traditions of holistic health that they have too often treated as folklore.

### 5.4 Theoretical contributions

The study advances three literatures. First, it extends the psychology of ritual from the laboratory to the life course, showing how ritual’s emotion-regulating, connecting, and meaning-making functions operate when ritual is sustained, collective, embodied, and biographically embedded, and identifying later life as a stage at which all of them are acutely needed (Hobson et al., 2018). Second, it contributes to the understanding of healthy ageing by demonstrating, in participants’ own accounts, how a single everyday practice can concurrently support physical vitality, cognition, emotion, belonging, and meaning, the dimensions usually studied and serviced in isolation and by offering an empirical, programme-based instantiation of gerotranscendence (Tornstam, 2005), a theory more often described than observed. Third, it contributes to cultural psychology by specifying how a holistic conception of the person, drawn from Chinese medical and ethical traditions, can be operationalized in a contemporary community programme, and by showing that such cultural grounding is a condition of effectiveness rather than a constraint upon it.

### 5.5 The tension between dependence and autonomy

One tension qualifies these findings. The programme’s belief work was anchored in a charismatic designer, and its emotional gravity was strong; participants spoke of the staff with intense attachment and of wanting them never to leave. This dependence had a clear function that it built trust and lent the new beliefs authority, but it was also a vulnerability. Especially in the follow-up interview, several participants doubted the group could sustain itself without the staff, and follow-up contacts over subsequent years indicated that the wider organization contracted and lost personnel. This illustrates a genuine dilemma: the charisma that catalyzes a ritual community can undermine the autonomous self-maintenance that ageing well ultimately requires (Ryan and Deci, 2000; Bandura, 1997). The programme’s “loose-but-firm” design—fixed core rituals combined with permission to adapt, skip, and lead, and its cultivation of service-user group leaders were partial, incomplete attempts to resolve this tension by transferring agency to participants. Future programes should treat the deliberate, staged handover of ownership to older adults not as an afterthought but as a core design principle, so that a comprehensive ritual practice becomes genuinely the community’s own.

### 5.6 Strengths and limitations

Strengths of the study include its multi-perspective sample spanning designer, coordinators, and service users; sustained engagement with the field after 6 years; an explicit conceptual framework; and a reflexive analytic process with documented attention to researcher positionality.

Several limitations should also be noted. First, the study was based on 60-min qualitative interviews with 21 participants aged 48–77 years. Although qualitative research prioritizes depth of understanding rather than statistical representativeness, the relatively small sample means that the findings may not reflect the experiences of the broader population of older adults. In particular, adults older than 77 years were not included, and this group may face different or more intensified challenges, such as declining health, greater dependency, mobility limitations, or social isolation.

Second, the findings rely on participants’ self-reported and retrospective accounts. We did not collect standardized physical or psychological measures, and participants’ narratives may have been shaped by recall bias, personal beliefs, or the social desirability of presenting themselves, their community, or a valued programme in a positive light. Therefore, the study identifies perceived meanings and plausible processes rather than measuring quantified programme effects.

Third, selection bias may have influenced the findings. The sample mainly included individuals who had remained involved in the programme and were willing to participate in interviews. Those who joined and later withdrew, declined to participate, or had less positive experiences are under-represented. Their accounts may have provided a more critical or complex picture, particularly regarding loneliness, barriers to participation, inadequate support services, financial difficulties, interpersonal conflicts, or tensions between dependence and autonomy.

Fourth, because the study focused on identifying the benefits of ageing within a community, negative or ambivalent experiences may have received less attention. Although participants did discuss challenges, the study design may have unintentionally foregrounded positive aspects of community ageing and underexplored difficulties related to exclusion, unmet care needs, or unequal access to support.

Fifth, the study concerns a single programme in one Chinese city. The specific social, cultural, institutional, and environmental characteristics of this setting shaped participants’ experiences and may limit the transferability of the findings to other contexts. Future research should examine whether similar processes occur in rural Chinese communities, other urban settings, and non-Chinese cultural contexts.

Finally, interviews were conducted in Mandarin and translated into English for reporting. Although translations were checked, some culturally specific meanings or nuances may have been attenuated during translation. Despite these limitations, the qualitative approach provides valuable insight into participants’ lived experiences and highlights factors that may support positive ageing in community-based environments.

### 5.7 Directions for future research

Four priorities follow. First, mixed-methods designs should pair this process-focused account with validated measures spanning the full range of benefit reported here, such as physical function, sleep, loneliness, perceived control, emotion regulation, meaning in life, and gerotranscendence, so that the comprehensiveness of the practice is matched by comprehensiveness of measurement (Reangsing et al., 2021; Yu, 2022). Second, longitudinal designs should test the temporal ordering implied by participants’ accounts, in which belonging and bodily-emotional engagement are entry points that make belief change and the cultivation of meaning possible. Third, research should examine for whom and under what conditions the radical internal-attribution belief (“*I am the source of everything*”) is empowering rather than self-blaming. Fourth, comparative work across ritual forms and cultural contexts, for example, square-dance collectives in China, communal practices in other ageing societies would help distinguish the culturally specific from the transferable elements of ritual-based support for ageing well.

## 6 Conclusion

Population ageing demands support for later life that is comprehensive, culturally meaningful, scalable, and low in cost. This study shows that a community-driven, ritual-based programme can support ageing well by working within a single, culturally grounded practice on the body, the mind, the emotions, social belonging, and the search for meaning together. Its value lies not in any one of these effects but in their integration: in offering older adults a dignified, familiar, holistic way of ageing rather than a clinical intervention aimed at a single deficit. Understood this way, community ritual is not a cultural residue to be tolerated but a sophisticated and transferable resource for an ageing world. Realizing its potential will depend on designing such programes so that the belonging, vitality, and meaning they generate are progressively handed to older adults themselves, so that a flourishing later life becomes, in the end, their own ritual and their own achievement.

## Data Availability

The raw data supporting the conclusions of this article will be made available by the authors, without undue reservation.
